# Wanted—A Rara Avis

**Published:** 1871-09

**Authors:** 


### Wanted—A Rara Avis.

A physician is wanted in a desirable location in the northern section of the State. The application calls for "a graduate of some respectable college, of good common sense and urbane manner—one not too airy or technical, or in the habit of boasting of the great practice he has had elsewhere, and in his cases and cures—

a man of temperate habits, and disposed to observe the recognized code of professional ethics." In drawing this picture, we fear that the writer has unwittingly described such rare attainments as are seldom united in one individual.—*Pacif. Med. Journal.*

